# Data on the effects of the anionic protein meal BioChlor^Ⓡ^ on sows before and after farrowing

**DOI:** 10.1016/j.dib.2023.109168

**Published:** 2023-04-20

**Authors:** David Henman, Ian John Lean, Elliot Block, Helen Marie Golder

**Affiliations:** a Riverlea Australia, Lot 411 Redlands Rd, Corowa, New South Wales 2646, Australia; b Scibus, 2 Broughton Street, Camden, New South Wales 2570, Australia; c Arm & Hammer Animal and Food Production*,* Princeton, New Jersey, 08540 USA

**Keywords:** Transition diet, Farrowing, Piglet survival, Reproduction

## Abstract

Three hundred and two parity 3 and 4 sows were allocated to one of three treatment groups: A (n=106): Control group fed the standard lactation diet; B (n=94): Lactation diet supplemented with 10 kg BioChlor/T; C (n=102): Lactation diet supplemented with 20 kg BioChlor/T. The sows were randomly allocated to treatment on entry to the farrowing shed at 100 d of gestation. The numbers allocated to each treatment were not equal with fewer sows allocated to treatment B at the start of treatment feeding than originally intended. Six allocated sows were not pregnant at their due farrowing date and two control group sows died after treatment feeding commenced prior to farrowing. All sows were individually housed in sow stalls and were fed 3 kg of their treatment diet once a day from d 105 of gestation. At d 110 of gestation, sows were moved into farrowing crates and continued to be fed 3 kg of their treatment diet once a day until the day of farrowing followed by *ad libitum* feeding of the treatment diet during a 27-d lactation. Approximately 50 litters from each treatment were randomly weighed to determine treatment effects on piglet average daily gain from birth to weaning. Litters were standardized within treatment to 10 piglets per litter at d 3 of lactation by allocating piglets from sows within treatment that had more than 12 piglets. After weaning, all sows were transported to a commercial module and mated on the first display of estrus. Sows were offered a common boar shed diet (13.8 MJ DE/kg; 170 g protein/kg; 9 g lysine/kg) *ad libitum* from weaning to mating. Following mating, all animals were fed 2.5 kg of a gestation diet (13.0 MJ DE/kg; 125 g protein/kg; 6 g lysine/kg) until farrowing. All sows were stalled individually during the gestation period following treatment feeding. Measures included: date of birth, number of piglets stillborn, number of piglets born alive, total number of piglets born, number of mummified feti, litter weight and number of piglets weighed at birth, litter weight and number of piglets at d 3, 14, and 26, number of piglets stillborn (gestation 2), number of piglets born alive (gestation 2), and total number of piglets born (gestation 2). The number of piglets born alive, number of total piglets born, and all weight measures were analyzed with mixed models with treatment as a fixed effect and sow within farrowing house as a random effect. A negative binomial model was used to estimate the incidence of still birth with sow within farrowing house as a random effect. For the odds of being re-mated a logistic regression mixed model was used to evaluate differences among treatment groups. These data provide information on an individual animal basis that can be used to inform pig producers, nutritionists, veterinarians, and researchers for further investigation on the use of anionic feeds in gestation diets of pigs and is suitable for future meta-analyses.

**Specifications Table**

**Table:** 

Subject	Animal Science
Specific subject area	The effects of an anionic protein meal fed 10 d before farrowing and during a 27-d lactation on piglet numbers and weights during farrowing and a second farrowing.
Type of data	Excel Spreadsheet and Tables
How the data were acquired	A lactation diet was fed to 302 parity 3 and 4 sows as a control (A; n=106) and either 10 kg/T (B; n=94) or 20 kg/T (C; n=102) of an anionic feed (BioChlor; Biovance USA) were added by hand and mixed through a feed mill. Sows were fed 3 kg of their respective diets/d from d 105 of gestation for 10 d to anticipated farrowing and then the same diets *ad libitum* for 27 d of lactation. Sows were fed a common diet (13.8 MJ DE/kg; 170 g protein/kg; 9 g lysine/kg) *ad libitum* from weaning to mating then 2.5 kg of a gestation diet (13.0 MJ DE/kg; 125 g protein/kg; 6 g lysine/kg) until farrowing.
Data format	Raw and analyzed.
Description of data collection	Sows identity, parity, mating date, and location were obtained from herd records. Data were collected as observations (farrowing, weaning, mating, and subsequent farrowing dates), count data (piglets – total born, mummified, stillborn, and alive at birth, d 3, d 14, weaning, and for subsequent farrowing), or weight data (litter weight, d 3, d 14, weaning, and subsequent litter) using pig scales.
Data source location	City: Corowa NSW Region: Riverina, Southern New South Wales Country: Australia Latitude and longitude: 35°55′53.71″ S 146°21′10.17″ E
Data accessibility	Repository name: figshare Data identification number: DOI:10.6084/m9.figshare.21994961 Direct URL to data: https://doi.org/10.6084/m9.figshare.21994961.v1 Instructions for accessing these data: None

## Value of the Data

• Pig producers, nutritionists, veterinarians, and researchers can benefit from using these data to provide the basis for further investigation of the use of anionic feeds in gestation diets of pigs.
• These data will be of interest to physiology investigators interested in the interactions of gestational diets with health and reproduction of mammalian species. In particular the data will help inform investigations into the role of anionic diets in influencing interactions among skeleton, energy, and protein metabolism.
• These data provide an indication of the inclusion rates for anionic protein meal intervention added to a standard lactating sow diet that may influence the incidence of stillbirth in sows and increase litter size at a subsequent farrowing.
• The study provided the rationale for a further larger study to evaluate the effects of anionic feeds in gestation of pigs.
• These data can be used to produce a meta-analysis with similar studies using individual animal observations to produce more definitive estimates of effects of pre-farrowing diets on piglet survival and performance of sows.

## 1 Objective

Observations in many species suggest that the adaptation to lactation is a period of substantial metabolic challenge for the periparturient animal. Calcium metabolism, in particular is challenged. In some species a negative cation-anion difference diet has improved the adaptation to birth and lactation. This randomised controlled clinical trial was designed to evaluate the responses of sows to a dietary intervention that would create a negative dietary cation-anion difference at farrowing and in the immediate post farrowing period. Our objectives overall were to assess two different concentrations of the anionic feed BioChlor on the performance of sows and their progeny. It was hypothesised that the negative dietary cation-anion difference would reduce the still birth of piglets and increase weight gains and weaned litter weight of piglets. The intention was to evaluate mortality of piglets in the current farrowing and to evaluate weight gain of the litters and to evaluate reproductive performance of the sows in the subsequent farrowing. We intended to provide farmers, animal scientists, veterinarians and other advisors with rigorous observations on the responses to anionic feeds of farrowing sows and to improve the health and productivity of sows and piglets.

## 2 Data Description

The raw data file and list of variable abbreviations are in [1]. The database contains raw data from 296 sows that were mated from November 1999 and were included in the final dataset. Of these sows 176 had a second farrowing. The dataset contains 46 columns of descriptors and variables.

Table 1 shows the ingredients fed in diets A, B, and C.

**Table 1:** Experimental diets: Diets A, B, and C were formulated using the following ingredients.

Ingredient (% as fed)	Diet A	Diet B	Diet C
Wheat	45.0	45.0	45.0
Barley	15.0	15.0	15.0
Lupin kernels	15.0	13.7	11.0
Mill mix	8.90	8.77	6.80
Rice pollard	5.00	5.00	7.50
Meat meal	6.00	6.00	6.00
Blood meal	1.00	1.03	1.03
Water	1.00	1.00	1.00
Tallow	1.5	1.87	2.20
Salt	0.35	0.35	0.35
Molasses	0.63	0.63	0.63
Lysine hydrochloride	0.32	0.32	0.32
DL-methionine	0.08	0.08	0.08
Threonine	0.13	0.13	0.13
Mineral premix	0.15	0.15	0.15
Virginiamycin (500 mg/kg)	0.01	0.01	0.01
Red micro grits	0.10		
Orange micro grits		0.10	
Violet micro grits			0.10
BioChlor^1^		1.00	2.00

1 Biovance USA.

Table 2 shows the effects of treatment on farrowing performance after feeding for 10 days prior to farrowing.

**Table 2:** Effects of anionic protein meal (BioChlor) supplementation to the lactation diet on farrowing performance after feeding for 10 days prior to farrowing. Numbers of litters reflect the number of sows farrowing.

Treatment	Number of litters	Number of piglets born alive	Total number of piglets born	Still birth (%)	Piglet birth weight (kg)
Diet A – control	104	10.7 ± 0.28	11.5 ± 0.31	7.2	1.50 ± 0.03^a^
Diet B - 10 kg/T	91	10.4 ± 0.30	11.3 ± 0.33	7.2	1.57 ± 0.03^b^
Diet C - 20 kg/T	101	10.8 ± 0.28	11.8 ± 0.31	7.4	1.53 ± 0.02^ab^
*P-*value		0.58	0.57	NA	0.09

Superscripts that share a letter are not significantly different at the 5% level.

NA – not applicable (descriptive only).

Table 3 shows the effects of treatment on weight of litters of sows in a randomly selected subpopulation of the population originally enrolled.

**Table 3:** Effects of an anionic protein meal (BioChlor) added to the lactation diet on weight of litters of sows in a randomly selected subpopulation of the population originally enrolled. Sows were offered diets *ad libitum* and piglets were weaned at 26.1 ± 0.1 days.

Treatment	Number of litters	Day 3 piglet weight (kg)	Day 14 piglet weight (kg)	Day 3 to wean average daily gain (kg/d)	Piglet average wean weight (kg)	Litter weight at weaning (kg)
Diet A – control	57	1.95 ± 0.04	4.76 ± 0.11	0.260 ± 0.01	7.85 ± 0.19	70.2 ± 2.21
Diet B - 10kg/T	54	1.99 ± 0.04	4.88 ± 0.11	0.265 ± 0.01	8.01 ± 0.20	71.0 ± 2.23
Diet C - 20kg/T	59	1.99 ± 0.04	4.89 ± 0.11	0.268 ± 0.01	8.23 ± 0.20	72.2 ± 2.22
*P-*value		0.80	0.58	0.55	0.16	0.80

Table 4 shows the effects of treatment on reproductive performance of sows and piglet numbers from gestation 2.

Table 5 shows the effects of treatment on risk of still birth in the first gestation (Gestation 1), odds of being remated for sows farrowing in gestation 1, and risk of still birth in the subsequent gestation (Gestation 2). The numbers of litters contributing to the relative risk of still birth are also indicated.

**Table 4:** Effects of an anionic protein meal (BioChlor) added to the lactation diet on reproductive performance of sows as indicated by interval from weaning to mating and offered diets *ad libitum*. Piglets were weaned at 26.1 ± 0.1 days. The numbers of piglets born alive, total born and stillborn are for the second gestation on study. Numbers of sows are for those that were mated a second time.

Treatment	Number of litters	Wean to mating (days)	Number of piglets born alive	Total number of piglets born	Stillborn (%)
Diet A - control	77	5.2 ± 0.51	11.2 ± 0.39	12.7 ± 0.42	12.1
Diet B - 10 kg/T	67	6.2 ± 0.53	11.1 ± 0.40	12.1 ± 0.42	8.3
Diet C - 20 kg/T	70	5.99 ± 0.54	11.7 ± 0.39	12.6 ± 0.42	6.2
*P-*value		0.31	0.44	0.51	NA

NA – not applicable (descriptive only).

**Table 5:** Effects of an anionic protein meal (BioChlor) added to the lactation diet on risk of still birth in the first gestation (Gestation 1), odds of being remated for sows farrowing in gestation 1, and risk of still birth in the subsequent gestation (Gestation 2). Numbers of litters contributing to the relative risk of still birth are indicated.

Treatment	Number of litters Gestation 1	Relative risk for still birth Gestation 1 (*P*-value)	Odds of being remated sows (*P*-value)	Number of litters Gestation 2	Relative risk for still birth Gestation 2 (*P*-value)^1^
Diet A – control	104	referent	Referent	77	Referent
Diet B - 10 kg/T	91	1.01 (0.96)	1.38 (0.30)	67	0.66 (0.05)
Diet C - 20 kg/T	101	1.03 (0.88)	1.20 (0.55)	70	0.55 (0.01)
*P-*value		0.99	0.58		0.02

1 Indicates that Diet B and Diet C differ from Diet A, but not each other.

## 3 Experimental Design, Materials and Methods

### 3.1 Study design

This was a completely randomized three treatment comparison in a one-way analysis of variance evaluation of the following diets: Control lactation; Control lactation with 10 kg/T BioChlor; Control lactation with 20 kg/T BioChlor. The unit of interest is the sow and unit of measurement the sow or piglet. The study was conducted at Bunge Proprietary Limited, Corowa NSW, Australia in 1999. 35°55′53.71″ S 146°21′10.17″ E. Three hundred and two commercial first-cross sows of parity 3 and 4 were enrolled at 100 days of gestation at entry to the farrowing house and randomly allocated to treatment: Enrollment was split over eight weeks such that 40 sows entered each week. There were 3 diets; diet A, a control lactation diet; diet B, the control lactation diet and 10 kg/T Biochlor; diet C, the control lactation diet and 20 kg/T Biochlor. The treatment period was for 6 weeks and was conducted between February to June 1999. Sows were medically treated if clinical disease was present.

The numbers allocated to each treatment were not equal with fewer sows allocated to treatment B at the start of treatment feeding than originally intended. Six allocated sows were not pregnant at their due farrowing date and two sows died after treatment feeding commenced prior to farrowing (both Control sows on Diet A). All sows were individually housed in sow stalls and fed 3 kg of their treatment diet once a day from gestation d 105. At d 110 of gestation, sows were moved into farrowing crates and continued to be fed 3 kg of treatment diet once a day until the day of farrowing. Sows were fed *ad libitum* treatment diet until d 27 of lactation.

After parturition, the number of piglets born alive, stillborn, and total litter size was recorded. Litters (born alive and stillbirths) were weighed and recorded as a birth litter weight. After two d, litters were weighed at the start of the lactation assessment period. Approximately 50 litters from each treatment were randomly weighed to determine treatment effects on piglet average daily gain from birth to weaning. Litters were standardized within treatment to 10 piglets per litter at d 3 of lactation and by moving piglets from sows with greater than 12 piglets to those with less than 8 piglets. Piglets were selected from the sows with the greatest litter sizes first. Litters were weighed at d 14 and at weaning at 26.8 ± 0.34 days of age. Sows were offered their treatment diet *ad libitum* during a 27-d lactation.

After weaning, all sows were transported to another building and mated on the first display of estrus with 3 × 10^9^ cells per dose of Large White × Landrace cross F1 semen and a second dose on the next morning. Animals were offered a common boar shed diet (13.8 MJ DE/kg; 170 g protein/kg; 9 g lysine/kg) *ad libitum* from weaning to mating. Following mating, all animals were fed 2.5 kg of a gestation diet (13.0 MJ DE/kg; 125 g protein/kg; 6 g lysine/kg) until farrowing. All sows were housed individually in sow stalls during the gestation period following treatment feeding. The numbers of piglets born alive, total born, and stillborn were recorded from this second farrowing.

### 3.2 Summary of measurements

The following measurements were made; date of birth, number of piglets stillborn, number of piglets born alive, total number of piglets born, number of mummified feti, litter weight and number of piglets weighed at birth, litter weight and number of piglets at d 3,14, and 26 of life, number of piglets stillborn (gestation 2), number of piglets born alive (gestation 2), and total number of piglets born (gestation 2).

### 3.3 Statistical analysis

All analyses were conducted using Stata (Version 17.1, College Station, Texas). For the following variables, number of piglets born alive, number of total piglets born, and all weight measures, a mixed models analysis (*mixed* Stata) was used with treatment as a fixed effect and sow within farrowing house as a random effect. Margins and standard errors were calculated post estimation (*margins* Stata) and pair-wise comparisons on the margins among diet groups made (*pwcompare* Stata). For estimates of still birth, a negative binomial model (*xtnbreg* Stata) was used to estimate the incidence with sow within farrowing house as a random effect. For the odds of being re-mated a logistic regression mixed model (*xtlogit* Stata) was used to evaluate differences among the treatment groups.

## Ethics Statements

This study complied with the National Institutes of Health guide for the care and use of laboratory animals (NIH Publications No. 8023, revised 1978) and ARRIVE guidelines.

## CRediT Author Statement

**David Henman:** Study protocol development, Methodology, and conduct in the field; **Ian Lean:** Conceptualization, Methodology, Statistical analysis, Editing, Visualization; **Elliot Block:** Review, Funding aquisition; **Helen Golder:** Editing, Data curation.

## Declaration of Competing Interest

The authors declare that they have no known competing financial interests or personal relationships that could have appeared to influence the work reported in this paper.

## Data Availability

Raw data on the effects of the anionic protein meal BioChlor® on sows before and after farrowing (Original data) (figshare).
